# Effects of *Kudoa septempunctata* genotype ST3 isolate from Korea on ddY suckling mice

**DOI:** 10.1051/parasite/2016020

**Published:** 2016-04-11

**Authors:** Yeounghwan Jang, Meejung Ahn, Hyojin Bang, Bongjo Kang

**Affiliations:** 1 Ocean and Fisheries Research Institute, Jeju Special Self-Governing Province Pyoseon-myeon, Segwipo-si Jeju 63629 Republic of Korea; 2 School of Medicine, Jeju National University Jeju 63243 Republic of Korea; 3 College of Veterinary Medicine, Jeju National University Jeju 63243 Republic of Korea

**Keywords:** *Kudoa septempunctata*, ST3 genotype, Food-borne disease, Myxozoa, *Paralichthys olivaceus*, suckling mice

## Abstract

This study investigated the effects of *Kudoa septempunctata* genotype ST3 spores on ddY suckling mice. Purified *Kudoa septempunctata* spores were administered into the stomachs of the mice at 5 × 10^6^ or 5 × 10^7^ spores/mouse, with inactivated *Kudoa* (5 × 10^6^ spores/mouse) or vehicle as controls. No abnormal clinical symptoms were observed and there were no variations in fluid accumulation ratio and cytokine gene expression in all groups. In addition, intact *Kudoa* spores and the 18S rDNA gene were only detected (by microscopy and quantitative PCR, respectively) in the groups administered such spores. This study thus confirms that spores from the ST3 strain of *Kudoa septempunctata* were excreted in the faeces without infecting the gastrointestinal tract in ddY suckling mice.

## Abstract (fr)

Cette étude a examiné les effets des spores de *Kudoa septempunctata*, de génotype ST3, sur des souriceaux à la mamelle de race ddY. Les spores purifiées de *Kudoa septempunctata* ont été administrées dans les estomacs des souris à 5 × 10^6^ ou 5 × 10^7^ spores/souris, avec comme témoins des *Kudoa* inactivés (5 × 10^6^ spores/souris) ou un véhicule. Dans tous les groupes, aucun symptôme clinique anormal n’a été observé et il n’y avait pas de variation dans les taux d’accumulation de fluide ni dans l’expression des gènes de cytokines. De plus, des spores de *Kudoa* intactes et le gène d’ADNr 18S de *Kudoa* ont été détectés uniquement (par microscopie et PCR quantitative, respectivement) dans les groupes auxquels ont été administrées ces spores. Cette étude confirme donc que les spores de la souche ST3 de *Kudoa septempunctata* ont été excrétées dans les fèces sans infecter le tractus gastro-intestinal chez les souriceaux ddY à la mamelle.

## Introduction

Fish and fishery products are an important source of nutrition for many millions of people worldwide. However, if such products are not handled and processed properly, consumers might suffer food-borne illnesses due to food-contaminating microbes such as bacteria, viruses, parasites and biogenic amines including histamine and biotoxins [5].

A total of 95 nominal species have been described in the myxosporean genus *Kudoa* (Kudoidae), which have been reported to infect the muscles, ovaries and intestines of marine teleosts [4, 10, 20]. Some species of the genus *Kudoa* can cause significant losses in the seafood industry either through direct mortality or by spoiling fish meat; that is, the infection often presents as either unsightly cysts dispersed in fish fillets or as postmortem myoliquefaction known as “jelly meat” [13].

Although myxozoan parasites are generally considered harmless to humans, certain human illnesses have been attributed to *Kudoa* spp., such as allergic symptoms [11]. The spores of *Kudoa septempunctata* Matsukane et al. 2010 [12] are composed of six or seven shell valves and polar capsules [12]. *Kudoa septempunctata-*infected olive flounders were strongly suspected of being associated with a diarrhoea outbreak, which was confirmed using ddY suckling mice [8]. The only proposed mechanism of infection was through the release of sporoplasm from ingested spores, which could reach the human intestine. The sporoplasm could then invade the human intestinal cell monolayer and cause severe damage [14].

Genotyping of *K. septempunctata* has shown that there are three different genotypes: ST1, ST2 and ST3. ST1 and ST2 types of *K. septempunctata* mostly parasitise flounders from Japan, while most of the ST3 strain has been found in flounder fish originating from South Korea [18]. The mitochondrial genome of *K. septempunctata* includes two genes namely cytochrome *c* oxidase subunit rRNA (*cox 1*) and the large subunit rRNA gene (*rnl*), a taxonomically important marker. Specifically, strain ST1 contains *cox 1*-1 and *rnl*-1 alleles; ST2 contains *cox 1*-2 and *rnl*-2 alleles; while ST3 contains *cox 1*-3 and *rnl*-2 alleles. These alleles differ in the *cox 1* and *rnl* genes by six and two single-nucleotide polymorphisms, respectively [18].

Studies have also assessed whether differences in the lineages within the species *K. septempunctata* are associated with food-borne illnesses and geographical origin [18]. In addition, the effects of *K. septempunctata* spores in mammals were assessed in adult BALB/c mice fed spores of *K. septempunctata* genotype ST3, which showed no pathological changes in the gastrointestinal tract despite detection of the *K. septempunctata* 18S rDNA gene in the stool samples of infected mice by quantitative PCR (qPCR) [1]. Also, recent studies with other myxosporeans, *Myxobolus honghuensis* spores exposed to BALB/c suckling mice, do not cause illness (diarrhoea and elevated fluid accumulation [FA]) and showed that *M. honghuensis* is not pathogenic for BALB/c suckling mice. [7].

One of the early responses of the host immune system to infection with protozoan parasites is the secretion of an array of potent cytokines, including tumour necrosis factor (TNF), interleukin 1 (IL-1) and IL-6 [17]. These cytokines act synergistically to prevent parasite survival by inducing production of specific T cells and antibodies against the parasite [19].

Clinical symptoms associated with parasite infection, including diarrhoea and a high fluid accumulation (FA) ratio, have been studied using suckling mice as they provide fast, reliable results [3]. Moreover, Kawai et al. [8] used ddY suckling mice to assess the pathogenicity of *K. septempunctata* spores and an associated food poisoning outbreak in Japan. Recently, Takeuchi et al. [18] classified three different strains of *K. septempunctata* with different pathogenicity for human digestion. Against this background, the present study was developed to clarify the pathogenicity of *K. septempunctata* ST3, a predominant strain in Jeju Island flounder fish, in ddY suckling mice using histological analysis, parasite enterotoxin tests, immune gene response and microscopic evaluation of stool samples.

## Materials and methods

### Animal model and ethics

Specific-pathogen-free (SPF) ddY mice were obtained from the Inasa Production Facility (SLC, Hamamatsu, Japan) and were bred in the Laboratory Animal Facility at Jeju National University (Jeju, South Korea). All animal experiments were carried out in accordance with the Jeju National University Guide for the Care and Use of Laboratory Animals (Permit Number: 2015-0013).

### Spore preparation

*Kudoa septempunctata*-infected olive flounder samples were collected from commercial fish farms located on Jeju Island. These fish were thoroughly investigated under a microscope at 400× magnification for the presence of cysts and the presence of *Kudoa* spores was confirmed by qPCR [8]. Conventional PCR was used to amplify two *K. septempunctata* mitochondrial genes: cytochrome c oxidase subunit I (*cox 1*) and large subunit rRNA (*rnl*) [18]. Negative controls (without template DNA) were included to check for contamination. The PCR products were sequenced on an ABI 3730XL DNA analyser. Mitochondrial genes were subjected to multiple sequence alignment using ClustalW (http://www.clustal.org) with MEGA v. 5.1.

Severe infection was defined as the presence of >10^5^ spores/g of tissue. Fish found to be severely infected were filleted for spore purification following the procedure of Ahn et al. [1] and the infected muscle samples were fixed in formalin for histopathological studies. Purified spores were diluted in phosphate-buffered saline (PBS) to final concentrations of 5 × 10^6^ and 5 × 10^7^ spores/100 μL. Some of the spore suspension was heated at 95 °C for 10 min to generate heat-treated *Kudoa* spores [8]. The viability of the purified spores was assessed by the trypan blue exclusion test [16].

### Suckling mouse experimental design

For the *K. septempunctata* spore infection experiment, about 165 suckling mice (4–5 days old), which had been separated from their mother 2 h prior to spore inoculation, were randomly divided into four groups as follows: 100 μL of PBS (*n* = 60) 5 × 10^6^ heat-treated *Kudoa* spores/100 μL (*n* = 35), 5 × 10^6^ *Kudoa* spores/100 μL (*n* = 60), and 5 × 10^7^*Kudoa* spores/100 μL (*n* = 10).

All mice were inoculated directly into the stomach using an oral injection needle and incubated at 25 °C throughout the experimental period (5 days). The mice were allowed to feed on their mother’s milk at 28, 56, 84 and 112 h post-ingestion (hpi) throughout the experimental period.

### Fluid accumulation (FA) ratio

At 0, 1.5, 3, 6, 9, 12 and 24 hpi, five mice from each group, except those receiving the higher dose of live spores, were sacrificed by cervical dislocation and their body weight was determined. The abdominal cavity was opened and the entire stomach and intestines were removed and blotted on absorbent paper. Adhering viscera and mesentery were removed, taking care to avoid breakage and subsequent fluid loss; then, the stomach and intestines were weighed. The FA ratio was determined as follows: weight of stomach plus intestines/(total body weight − weight of stomach plus intestines) [2].

### Bowel movements

The influence of *K. septempunctata* spores on the bowel movements of suckling mice was evaluated in ten mice from each group, except those given heat-treated spores. Briefly, each mouse was isolated in a separate cage each fitted with filter paper over the cage floor at 25 °C and monitored for 24 hpi. Bowel movements were evaluated by calculating the ratio between the number of faecal samples observed and the number of tested mice. The form of the stool on the filter paper was also recorded. Faeces collected after a bowel movement were checked by a wet smear to observe the presence of spores. The time course of the appearance of spores in faeces and the integrity of spores after passing through the alimentary tract of the mice were recorded [7].

### Quantitative PCR and immune gene expression analysis

For the immune gene expression analysis, sample tissues were collected from the small intestine from five mice each from the PBS and lower-dose live spore groups at 6, 12, 24, 48, 72 and 120 hpi; they were then subjected to RNA extraction using the RNeasy Plus Mini Kit (Qiagen, Hilden, Germany) and cDNA synthesis with Superscript III Reverse Transcriptase (RT; Invitrogen, Waltham, MA, USA), following the manufacturers’ instructions. The transcripts of proinflammatory (TNFα, IL-6) cytokine genes were measured by qPCR for immune system genes such as TNFα, IL-6 and the internal control gene β-actin using SYBR Green, following previously described methods [15]. The results were analysed using MX Pro-Mx3000P Multiplex Quantitative PCR System software (Stratagene, La Jolla, CA, USA) and the relative expression ratio (R) of mRNA was calculated using the formula 2^_ΔΔCt^ = 2^_(ΔCt (test)_ ΔCt (β-actin))^ [9]. Real-time PCR efficiency was determined by amplification of a dilution series of cDNA according to the equation 10^(−1/slope)^, which revealed consistency between the target genes and *β*-actin. The results are presented as means and standard deviations (SD).

### qPCR detection of *K. septempunctata*

Real-time qPCR was used to detect *K. septempunctata* 18S ribosomal DNA [8]. DNA was extracted from various organs and the stool suspension filtrates of five mice each from the PBS and lower-dose live spore groups using a QIAamp DNA Mini Kit (Qiagen, Venlo, Netherlands), following the manufacturer’s instructions. qPCR was performed using TaqMan Universal PCR Master Mix (Applied Biosystems, Carlsbad, CA, USA) with primer, qPCR probe and thermal conditions set as described by Kawai et al. [8]. The gene copy number was determined using the standard curve method with plasmid DNA containing a copy of the target gene as a control. Negative controls (without template DNA) were included to check for contamination.

### Histological studies

For histopathological analysis, various organs including the intestines were obtained from mice in all groups, except those given heat-inactivated spores. Muscle tissue of *Kudoa* spore-infected olive flounders was fixed in 10% neutral buffered formalin for paraffin embedding. The paraffin-embedded tissues were cut at a thickness of 5 μm using a rotary microtome (Leica, Nussloch, Germany). Tissue sections were then stained with haematoxylin and eosin.

### Statistical analyses

The mean ± SD of the assayed parameters was calculated for each group. Two-sample Student’s *t*-tests were used to compare values between individual experimental and control groups. Differences were considered significant at *P* < 0.05.

## Results and discussion

**Figure 1.**
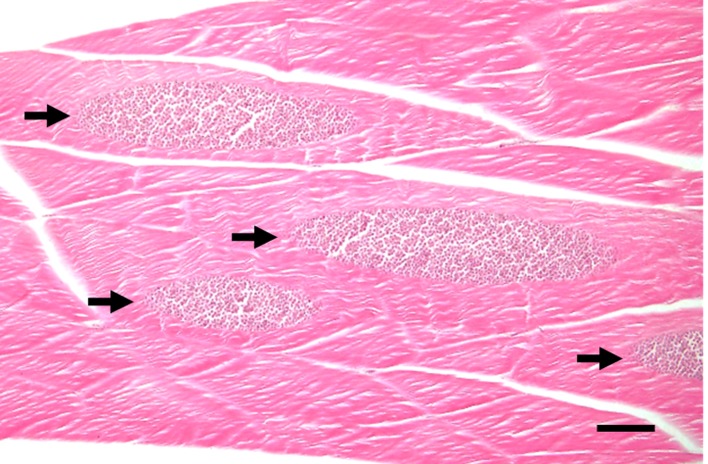
Stained histological section of olive flounder (*Paralichthys olivaceus*) muscle showing the muscle fibres substituted with *Kudoa* spores. Arrows indicate infection with *K. septempunctata* spores. Haematoxylin and eosin staining. Scale bar = 100 μm.

*Kudoa septempunctata* isolated from infected olive flounder muscle tissues collected from fish farms on Jeju Island were subjected to microscopic and sequence analyses to determine the strain. Figure 1 shows a histological section of infected flounder fish muscle tissue containing *Kudoa* spores without any other clinical symptoms such as inflammation and fibrosis (Fig. 1). In addition, purified *Kudoa* spores were confirmed to contain 6 or 7 shell valves and a polar capsule per spore (Fig. 4A), consistent with a previous report [1]. Sequence analysis of the mitochondrial genes *cox 1* and *rnl* revealed that the isolated *K. septempunctata* spores were of the ST3 genotype [18], as they harboured *cox 1*-3 and *rnl*-2.

**Figure 2.**
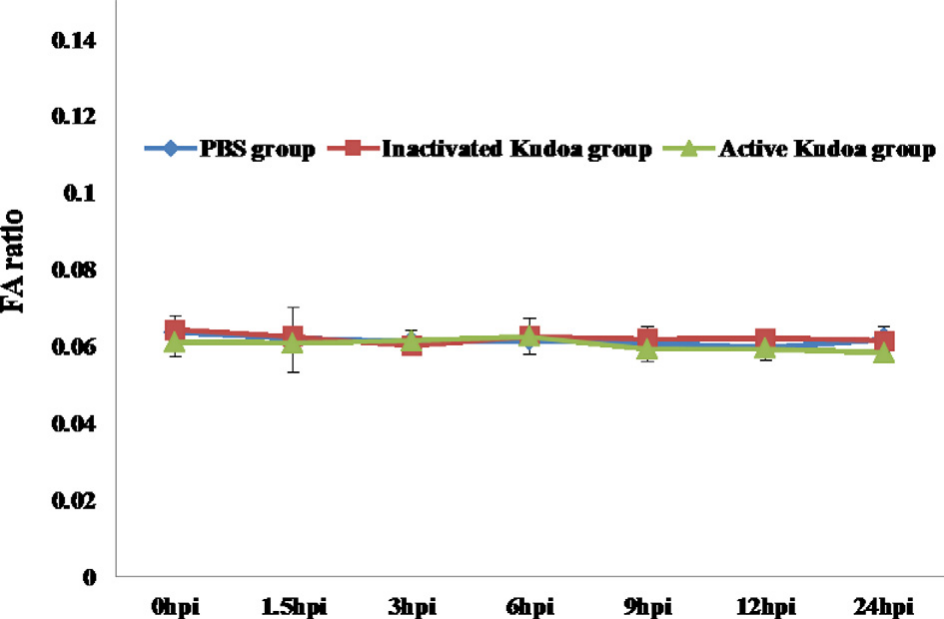
Kinetics of the fluid accumulation (FA) ratio in ddY suckling mice in phosphate-buffered saline, 5 × 10^6^ inactivated spores/mouse and 5 × 10^6^ active spores/mouse groups. Means of FA ratios ± standard deviation for five mice are shown. No significant differences (*P* < 0.05) were found at any time interval. Hpi: hours post-ingestion.

The three mouse groups orally administered *Kudoa* spores were monitored continuously for 24 h. No significant variations (*P* < 0.05) in FA ratios were observed among them throughout the incubation period (Fig. 2). Among these three mouse groups, the FA ratio of the group injected with active *Kudoa* spores was 0.060 ± 0.003, and that of the group with inactivated *Kudoa* spores was 0.062 ± 0.003, while that of the PBS-injected mouse group was 0.061 ± 0.002 (Fig. 2). Ratios of less than 0.070 are considered to be negative, so the *Kudoa* spores did not affect bowel movements of the infected suckling mice [3]. The FA ratio directly quantifies the diarrhoeal response of mice to an oral challenge with any enterotoxin. In a previous study, this method was used as a rapid screening procedure to determine the diarrhoea-inducing ability of cholera strains using infant mice [2].

**Figure 3.**
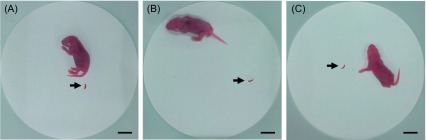
Bowel movements of suckling mice given PBS (A) or either dose of live spores (B and C) after 24 h of incubation at 25 °C. Each arrow represents faeces. Scale bars = 10 mm.

In addition, all groups had similar rates of bowel movements as the ratio of the number of faecal samples observed to the number of test mice in each group ranged between 0.8 (PBS and higher-dose spores) and 0.9 (lower-dose live spores) (Table 1; Fig. 3). Furthermore, no abnormal bowel movements such as watery stools or pasty discharges were recorded. A previous study recorded similar results of no abnormal bowel movements and no variation in FA ratios in suckling mice inoculated with *Myxobolus honghuensis,* a myxosporean parasite [7].

**Table 1.** Bowel movement results in suckling mice in the groups with phosphate-buffered saline (PBS) or *K. septempunctata* at 5 × 10^6^ or 5 × 10^7^ spores/mouse inoculated at 24 hpi.

Group	Body weight (g)	No. of faecal samples/tested mice	Watery stool form/inoculated mice
PBS	2.789 ± 0.355	0.8 (8/10)	0/10
5 × 10^6^ spores/mouse	2.744 ± 0.364	0.9 (9/10)	0/10
5 × 10^7^ spores/mouse	2.597 ± 0.287	0.8 (8/10)	0/10

FA ratio: fluid accumulation ratio, calculated from all of the tested animals in each group. Hpi: hours post-ingestion.

**Figure 4.**
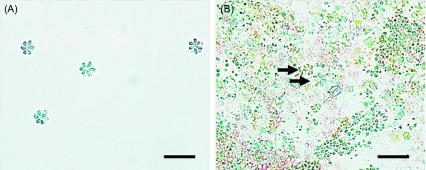
Unstained faecal wet smear from suckling mice orally administered *Kudoa septempunctata* spores. (A) Phase contrast micrograph of *K. septempunctata* spores in inoculation solution. (B) *Kudoa* spores detected in faeces 12 h post-ingestion. Scale bar = 20 μm.

Faeces from individual mice in each group were collected and checked for the presence of *Kudoa* spores in a wet smear using a microscope. Intact *K. septempunctata* spores were found only in faeces from the live *Kudoa* spore-inoculated groups (Fig. 4B), while no spores were recorded in the PBS-inoculated group. A previous study reported that the mechanism of infection of *K. septempunctata* is through the release of its sporoplasm, which plays an important role in mediating cellular toxicity [14]. However, in the present study, microscopic observation of intact *Kudoa* spores with qPCR detection for the 18S ribosomal DNA gene were observed only in faecal samples from mice infected with live spores from 6 hpi (*C*_t_ value: 37.74) until 24 hpi (*C*_t_ value: 43.68), similar to another recently published report [1].

**Figure 5.**
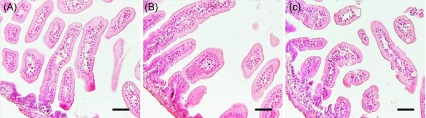
Representative figures of the small intestines of suckling mice inoculated at 24 h post-infection with phosphate-buffered saline (A), *K. septempunctata* at 5 × 10^6^ spores/mouse (B) and *K. septempunctata* at 5 × 10^7^ spores/mouse (C). A–C: Haematoxylin and eosin staining. Scale bars = 50 μm.

Microscopic study of intestinal tissues showed no pathological changes (Fig. 5). Mice given PBS showed intact intestinal sections with a normal appearance of villi and no pathological changes, as shown in Figure 5A. Similarly, those infected with either dose of live spores also showed intact intestinal villi without any inflammation or necrosis, even at 24 hpi (Figs. 5B and 5C). This study confirms that *Kudoa septempunctata* has no pathogenic effects on ddY suckling mice.

**Figure 6.**
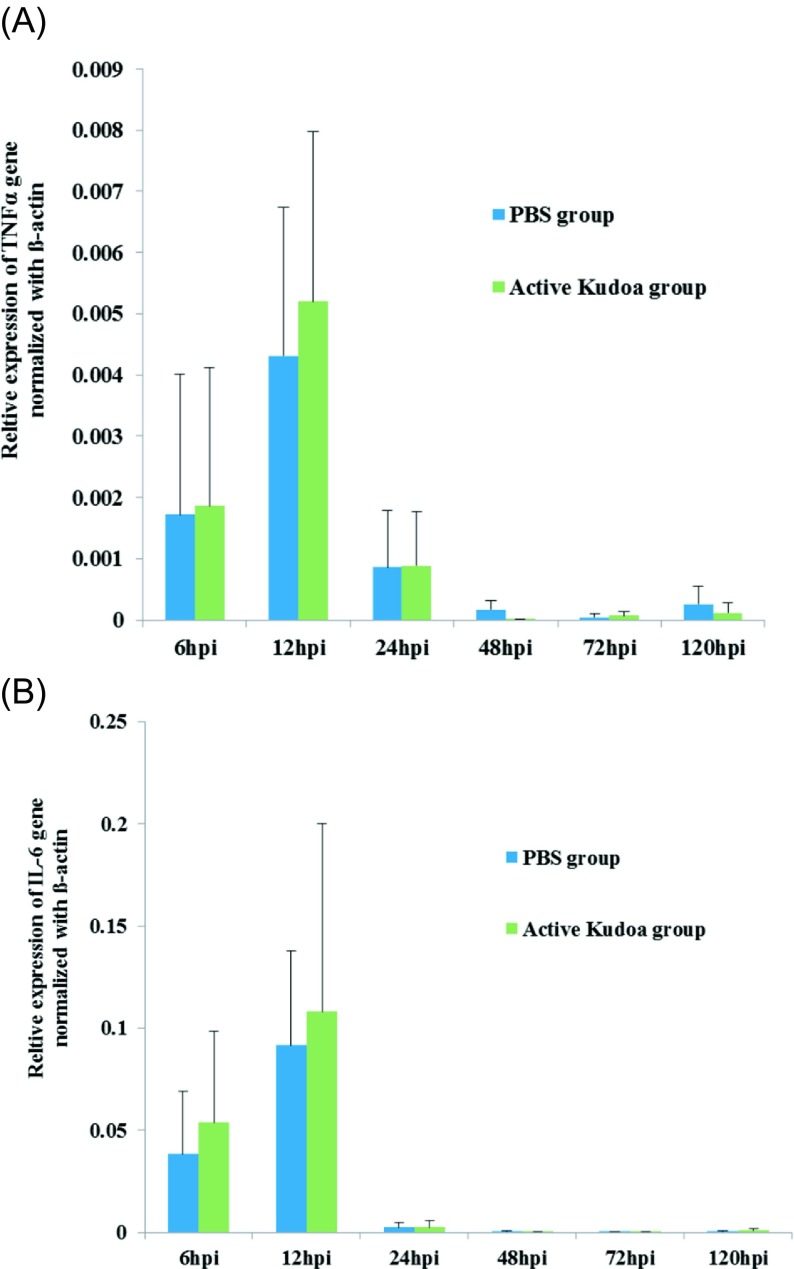
Induction of proinflammatory cytokines was assessed using the administration of phosphate-buffered saline or *K. septempunctata* at 5 × 10^6^ spores/mouse in suckling mice. Tumour necrosis factor (TNFα) (Fig. 6A) and interleukin 6 (IL-6) (Fig. 6B) expression levels were examined by SYBR green quantitative polymerase chain reaction and normalised using *β*-actin expression as an internal control. Relative levels of immune gene mRNA were analysed by the 2^−ΔCt^ method (the *C*_t_ value of the target immune gene minus the *C*_t_ value of the *β*-actin gene). Data are presented as means ± standard deviation. Hpi: hours post-ingestion.

The expression of proinflammatory cytokines, namely, TNFα and IL-6, was measured by real-time RT-PCR (Fig. 6). The expression kinetics of cytokines in all three suckling mouse groups indicated slight elevation in TNFα (Fig. 6A) and IL-6 (Fig. 6B) mRNA transcript levels at 6 through 12 hpi, which then dropped at 24 hpi, in PBS-treated mice and those given the lower dose of live spores. These results confirm the non-pathogenic nature of *Kudoa* spores in suckling mice, as a previous study on mice treated with *Trichinella spiralis* has shown dose-dependent immune gene expression [6], whereas in the present study, no variation could be found between the control and *Kudoa-*inoculated mouse immune gene expressions. Collectively, the findings in this study suggest that *K. septempunctata* strain ST3 from Jeju Island, South Korea, does not cause diarrhoea in ddY suckling mice. However, detailed studies including the other *K. septempunctata* strains (ST1 and ST2) might be required to further test previous reports of *K. septempunctata* spores as a human pathogen.

## Conflict of interest

There is no conflict of interest.
